# Synthesis of Rare-Earth-Doped Strontium Tungstate Phosphor at Room Temperature and Applied Flexible Composite

**DOI:** 10.3390/ma15248922

**Published:** 2022-12-13

**Authors:** Jung-Hyun Wi, Jae-Yong Jung, Sang-Geon Park

**Affiliations:** 1 Department of Smart Manufacturing Engineering, Changwon National University, Changwon 51140, Republic of Korea; 2 Research and Business Development Foundation, Engineering Building, Silla University, Busan 45985, Republic of Korea; 3 Department of Mechatronics Convergence Engineering, Changwon National University, Changwon 51140, Republic of Korea

**Keywords:** SrWO_4_, phosphor, luminescence, flexible

## Abstract

In this study, we successfully synthesized rare-earth-doped crystalline SrWO_4_ at room temperature by co-precipitation. The results from the X-ray diffraction analysis showed a main diffraction peak related to the (112) plane. Phosphors doped with either Dy^3+^ or Sm^3+^ ions showed strong light absorption in the UV region and blue-yellow and red light emission. To synthesize a white light phosphor, Dy^3+^ and Sm^3+^ ions were co-doped to produce a SrWO_4_:[Sm^3+^]/[Dy^3+^] phosphor. When the Sm^3+^ ion concentration was increased and the Dy^3+^ concentration was maintained, the red light intensity increased while the blue-yellow light intensity decreased. The composites were combined with polydimethylsiloxane (PDMS), and a flexible composite material was fabricated. The composite exhibited various luminescence properties under UV and visible light, which suggested its potential for use as an LED color filter.

## 1. Introduction

Phosphors doped with rare earth (RE) tungstates have attracted significant research interest because they can be used as white-light-emitting diodes, solid-state lighting, displays, and in various lighting industries [1,2]. Tungstates such as barium tungstate (BaWO_4_), strontium tungstate (SrWO_4_), and calcium tungstate (CaWO_4_) are chemically stable, have a broad absorption wavelength in the UV–vis region, and are capable of excellent energy transfer to rare earth ions [3,4,5]. Manufacturing a phosphor that emits a variety of colors with high luminescence intensity requires a thermally and chemically stable host structure doped with rare earth ions such as terbium (Tb^3+^), europium (Eu^3+^), dysprosium (Dy^3+^), and samarium (Sm^3+^), which possess many energy levels in the visible light region [6,7]. Among these rare earth ions, Dy^3+^ emits blue light via magnetic dipole transition (^4^F_9/2_→^6^H_15/2_) and yellow light via electric dipole transition (^4^F_9/2_→^6^H_13/2_) [8]. Sm^3+^ emits orange light via magnetic dipole (^4^G_5/2_→^6^H_7/2_) transition and red light via electric dipole transition (^4^G_5/2_→^6^H_9/2_) [9]. Hence, Dy^3+^ and Sm^3+^ are both compelling doping candidates for use in phosphor materials. Yu et al. synthesized a Ca_3−x_Li_x_(PO_4_)_2−x_(SO_4_)_x_:Dy^3+^, Sm^3+^ phosphor by the sol–gel method and investigated the effect of Sm^3+^ ion concentration on the crystal structure and luminescence intensity. The authors found that the co-doping of Dy^3+^ and Sm^3+^ emitted warm white light and proposed that it was an effective method to implement [10]. Jung et al. synthesized BaWO_4_ and CaWO_4_ using the co-precipitation method and doped Tb^3+^ and Eu^3+^ rare earth ions to produce green and red phosphors that can impart luminescent properties to the host [11,12]. Tan et al. synthesized a NaLa(WO_3_)_2_ phosphor doped with Dy^3+^ and Sm^3+^ using the thermal decomposition method. They found that when the doping concentration of Sm^3+^ increased, the intensity of the light emitted by Dy^3+^ decreased as energy was transferred from Dy^3+^ to Sm^3+^ [13]. Sun et al. also reported a color change due to energy transfer by the co-doping of Dy^3+^ and Sm^3+^ into Ca_3_TeO_6_ host crystals using the solid-state reaction method [14].

Herein, we report a successful synthesis of SrWO_4_ by a co-precipitation method at room temperature. We investigated the effect of doping SrWO_4_ with either Dy^3+^ or Sm^3+^, and co-doping SrWO_4_ with both Dy^3+^ and Sm^3+^ as well as the effect of altering the Sm^3+^ concentration. This study also characterized the luminescence intensity, crystal structure, and particle shape of the synthesized phosphors. Finally, a flexible LED filter was produced by combing the synthesized phosphor with a polymer.

## 2. Materials and Methods

### 2.1. Synthesis of the Crystalline SrWO_4_ and SrWO_4_:RE^3+^ by Co-Precipitation

Starting materials: Strontium acetate ((CH_3_CO_2_)Sr, Sigma-Aldrich), sodium tungstate (Na_2_WO_4_·2H_2_O, Sigma-Aldrich), dysprosium nitrate (Dy(NO_3_)_3_·xH2O, Dy^3+^, Sigma-Aldrich), and samarium nitrate (Sm(NO_3_)_3_·6H_2_O, Sm^3+^, Sigma-Aldrich).

**Figure 1:**
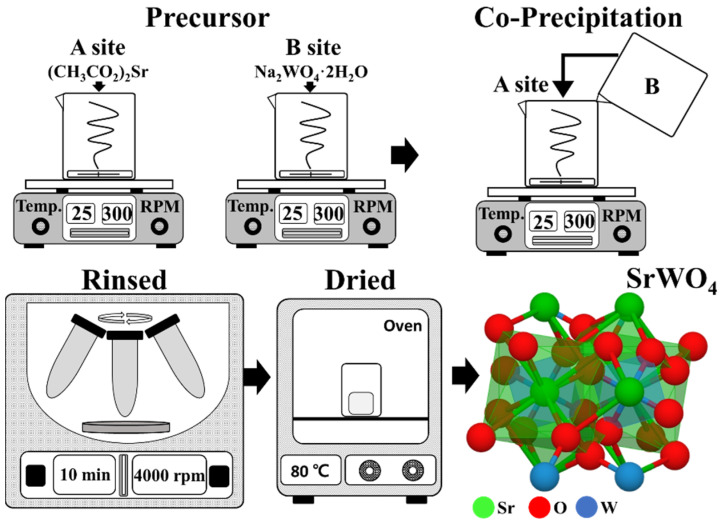
Schematic of co-precipitation procedure.

A total of 1 mmol of (CH_3_CO_2_)Sr and 1 mmol of Na_2_WO_4_·2H_2_O were placed in beakers ‘A’ and ‘B’, respectively, and each beaker was combined with 100 mL of distilled water. The solutions in each beakers ‘A’ and ‘B’ were completely dissolved until the mixtures were transparent in color. Next, the two solutions were combined and stirred for 30 min at ambient temperature. After mixing, the final mixture changed to an opaque white color and formed a powder precipitate. The mixture was centrifuged at 4000 rpm to recover the powder. The recovered powder was washed twice with distilled water to remove side products and unreacted compounds and then centrifuged again. Finally, the powder was dried at 80 °C in an oven for 16 h (Figure 1). The phosphor was synthesized by adding 0.25 mmol each of Dy^3+^ and Sm^3+^ to beaker ‘A’, and the remaining steps were performed as above. White light phosphor was synthesized by co-doping Dy^3+^ and Sm^3+^ and fixing the amount of Dy^3+^ while altering the amount of Sm^3+^. The doping percentages for all samples are shown in Table 1.

### 2.2. Fabricated Flexible Color Composite

To prepare the composite, 0.1 g of the synthesized powder, 2 g of polydimethylsiloxane (PDMS) polymer, and 0.2 g of curing agent were mixed until homogenous. Next, the mixture was poured into a square mold and placed in an oven at 80 °C for 1 h to form the composite. The composite was then irradiated with an ultraviolet lamp to observe any color change.

### 2.3. Characterization

X-ray diffraction analysis (XRD) (X’Pert PRO MPD, 40 kV, 30 mA, Cu–Kα radiation (wavelength: 1.5406 Å)) was used to characterize the crystal structure of the synthesized phosphor powder. The XRD analysis was carried out at a scan rate of 4° per minute at a diffraction angle of 10° to 70°. The crystal grains’ size and microscopic surface shape were characterized with a scanning electron microscope (CZ, MIRA I LMH, TESCAN), and a fluorescence photometer (FS-2, Scinco) with a xenon lamp as a light source was used for emission and absorption characteristics.

## 3. Results & Discussion

### 3.1. Characteristics of SrWO_4_ and Single Doped SrWO_4_

**Figure 2:**
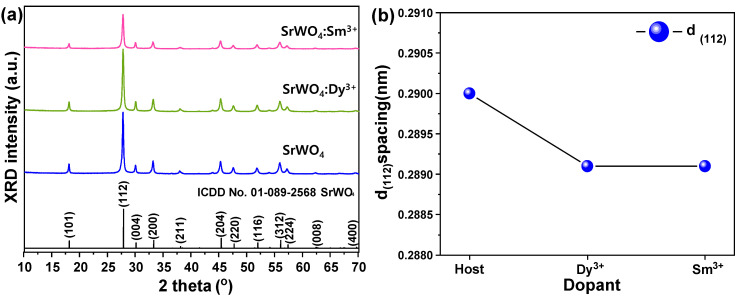
(**a**) XRD patterns of crystalline SrWO_4_ and SrWO_4_:RE^3+^ and (**b**) d_(112)_ spacing of samples.

Figure 2a shows the XRD peaks of SrWO_4_, SrWO_4_:Dy^3+^, and SrWO_4_:Sm^3+^. SrWO_4_ showed a tetragonal (a = 5.400 Å, b = 5.400 Å, c = 11.910 Å) structure that was in good agreement with ICDD # 01-089-2568. The single-doped samples used 0.25 mmol of rare earth ions. The XRD analysis showed a main diffraction peak on the (112) plane. Likewise, the RE-doped SrWO_4_ samples exhibited a strong (112) main peak.

**Figure 3:**
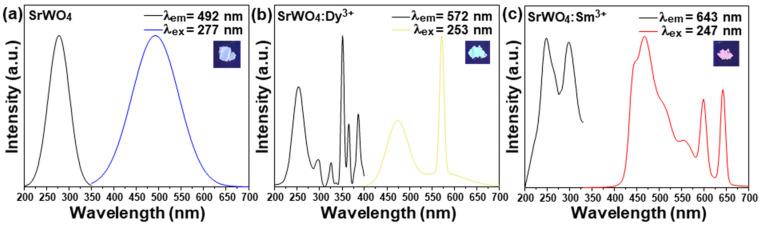
(**a**) Luminescence spectra of SrWO_4_, (**b**) SrWO_4_:Dy^3+^, and (**c**) SrWO_4_:Sm^3+^.

Figure 2b shows the lattice constant change with and without RE doping along the (112) plane. The lattice constant of the (112) plane significantly changed due to the RE doping (SrWO_4_: 0.290 nm, SrWO_4_:Dy^3+^: 0.2891 nm, SrWO_4_:Sm^3+^: 0.2891 nm). This change in the crystal lattice of SrWO_4_ by RE doping was attributed to the relatively large ionic radius (Sr: 1.18 Å, W: 0.66 Å, Dy: 1.07 Å, Sm: 1.22 Å) of the RE dopants [15]. Figure 3 shows the absorption and emission spectra of each sample. The host SrWO_4_ showed absorption from 220 nm to 340 nm and peaked at 277 nm. When the sample was excited at the highest peak (277 nm), a spectrum with a range of 350–650 nm peaking at 492 nm in a blue-white emission spectrum was observed (Figure 3a). Figure 3b shows the absorption and emission spectra of the SrWO_4_:Dy^3+^ phosphor powder. In the absorption spectrum controlled with an emission wavelength of 572 nm, the charge transfer band (CTB) absorption signal generated between the Dy^3+^ cations and O^2-^ anions peaked at 253 nm. Moreover, several absorption narrow bands generated within the 4f-4f electron arrangement of Dy^3+^ ions were observed [16]. Among them, the absorption wavelength at 351 nm had the strongest absorption intensity and signaled the ^6^H_15/2_→^6^P_7/2_ transition of Dy^3+^. The absorption wavelengths at 325, 364, and 386 nm, which had relatively weaker absorptions, were identified as ^6^H_15/2_→^6^P_3/2_ transitions. These absorption signals were generated by the ^6^H_15/2_→^6^P_5/2_ and ^6^H_15/2_→^4^I_13/2_ transitions [17]. After excitation at 253 nm, the SrWO_4_:Dy^3+^ phosphor powder exhibited a yellow emission spectrum with a peak at 572 nm due to the ^4^F_9/2_→^6^H_13/2_ electric dipole transition of Dy^3+^ ions. Furthermore, the blue emission band belonging to the host was also observed. Since the emission peak was strong due to the electric dipole transition, the Dy^3+^ ion doped in the SrWO_4_ host lattice was in a non-inversion symmetric site [18]. For the phosphor doped with Sm^3+^ ions, when the emission wavelength was controlled at 643 nm, a CT band with a peak at 248nm and an absorption band with a peak at 297nm were observed. This absorption signal originated from the Sm^3+^ ions located within the host lattice. When the synthesized sample was excited at 248 nm, blue light emission by the SrWO_4_ matrix, green light emission at 560 nm (^4^G_5/2_→^6^H_15/2_), orange light emission at 599 nm (^4^G_5/2_→^6^H_9/2_), and red light emission signals at 643 nm (^4^G_5/2_→^6^H_11/2_) were generated [19]. Since the intensity of the red emission caused by electric dipole transition was about 1.1 times stronger than that caused by magnetic dipole transition, the Sm^3+^ ions in the SrWO_4_ host crystal were located in non-inversion symmetric sites [20]. In addition, as in the undoped SrWO4 sample, blue light emission was observed in the doped specimen due to absorption and emission by the matrix in a wide range of light absorptions.

**Figure 4:**
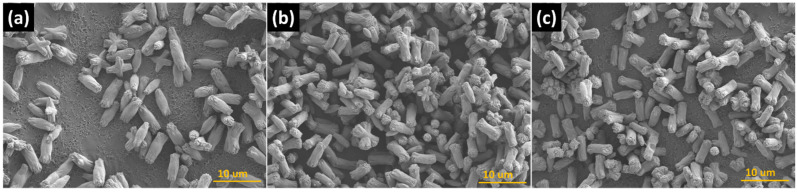
FE-SEM images of (**a**) SrWO_4_, (**b**) SrWO_4_:Dy^3+^, and (**c**) SrWO_4_:Sm^3+^ powders.

The FE-SEM images of all samples showed that they all had long cylindrical shapes. For SrWO_4_, the particle size was ~6.2 µm and ~1.92 µm in the longitudinal and transverse direction, respectively (Figure 4a). For the RE-doped SrWO_4_:Dy^3+^, the particle size averaged ~5.09 µm (longitudinal) and ~1.49 µm (transverse) (Figure 4b), and for SrWO_4_:Sm^3+^, the particles measured ~5.61 µm (longitudinal) and 1.49 µm (transverse) (Figure 4c).

### 3.2. Characteristics of [Sm^3+^]/[Dy^3+^] Co-Doped SrWO_4_

**Figure 5:**
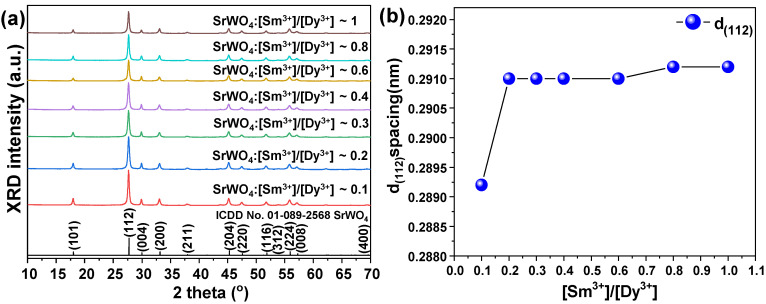
(**a**) XRD patterns of crystalline SrWO_4_:[Sm^3+^]/[Dy^3+^] and (**b**) d_(112)_ spacing of samples.

Figure 5a shows the XRD peaks of SrWO_4_ co-doped with the rare earth ions Dy^3+^ and Sm^3+^ for synthesis as a white-light-emitting phosphor. The XRD pattern did not indicate a secondary phase caused by the RE doping but exhibited the diffraction signal of the main peak (112). Figure 5b shows the lattice constant change in the (112) plane, which was the main peak of the RE-co-doped SrWO_4_:[Sm^3+^]/[Dy^3+^] samples. As reported above, the lattice constants of the SrWO_4_:Dy^3+^ and SrWO_4_:Sm^3+^ samples decreased compared to the un-doped sample. However, as seen in Figure 5b, the lattice constants of the SrWO_4_:[Sm^3+^]/[Dy^3+^] sample increased. The higher lattice constants could have been caused by crystal lattice distortion or structural change induced by the additional amount of RE ions having a rather large ionic radius.

**Figure 6:**
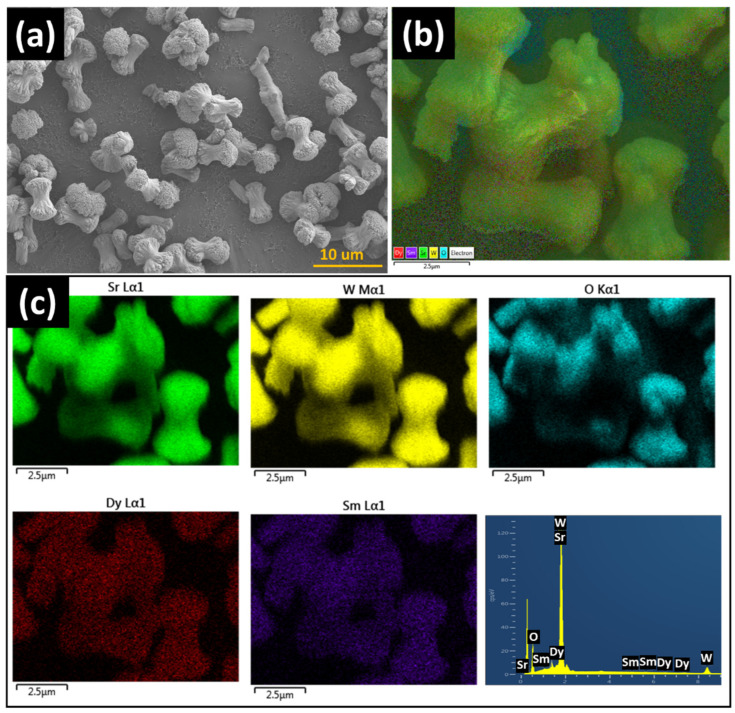
(**a**) FE-SEM image of SrWO_4_:[Sm^3+^]/[Dy^3+^], (**b**) EDS layered image, and (**c**) elemental mapping analysis.

Figure 6 shows the FE-SEM image and EDS mapping component analysis of the synthesized SrWO_4_:[Sm^3+^]/[Dy^3+^] phosphor. The particle shape grew in the longitudinal direction of a cylindrical shape and resembled a dumbbell, with an estimated size of 5.26 µm and 2.33 µm in the longitudinal and transverse directions, respectively. The EDS component analysis showed the presence of Sr, W, O, Dy, and Sm, which confirmed that the RE ions were doped (Figure 6b,c).

**Figure 7:**
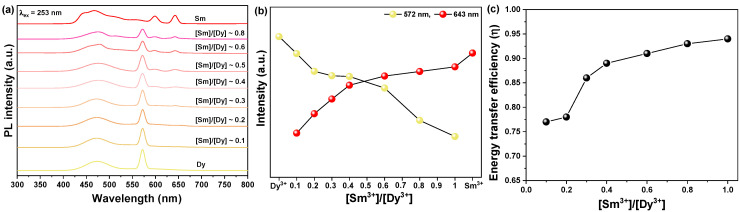
(**a**) PL spectra of SrWO_4_:[Sm^3+^]/[Dy^3+^] under 253 nm, (**b**) change in PL intensity at 572 nm and 643 nm, and (**c**) energy transfer efficiency.

Figure 7a shows the emission spectrum of the SrWO_4_:[Sm^3+^]/[Dy^3+^] phosphor powder co-doped while changing the Sm^3+^ ion concentration (the Dy^3+^ ion concentration was fixed). When excited at 253 nm and as the doping concentration of Sm^3+^ increased, the following were simultaneously observed: blue light at 492 nm, yellow light at 572 nm, orange light at 599 nm, and red light at 643 nm. As the concentration of Sm^3+^ ions increased, the intensity of yellow light emission by the Dy^3+^ ions decreased. The lower yellow light emission intensity meant that the emission energy was converted from the Dy^3+^ ions in the host lattice to the Sm^3+^ ions (Figure 7b). The energy transfer efficiency from the Dy^3+^ to Sm^3+^ ions can be expressed by Equation (1) [21].

(1) $$\;\;\eta =1-I/I_{0}$$

where *I* is the emission intensity of Dy^3+^ ions in SrWO_4_:[Sm^3+^]/[Dy^3+^] phosphors and *I*_0_ is the emission intensity of Dy^3+^ ions in the SrWO_4_:Dy^3+^ phosphors. As shown in Figure 7c, the energy transfer efficiency tended to increase as the amount of Sm^3+^ ions added increased. However, as the emission intensity decreased, a concentration-quenching phenomenon due to excessive rare earth doping was observed [22].

**Figure 8:**
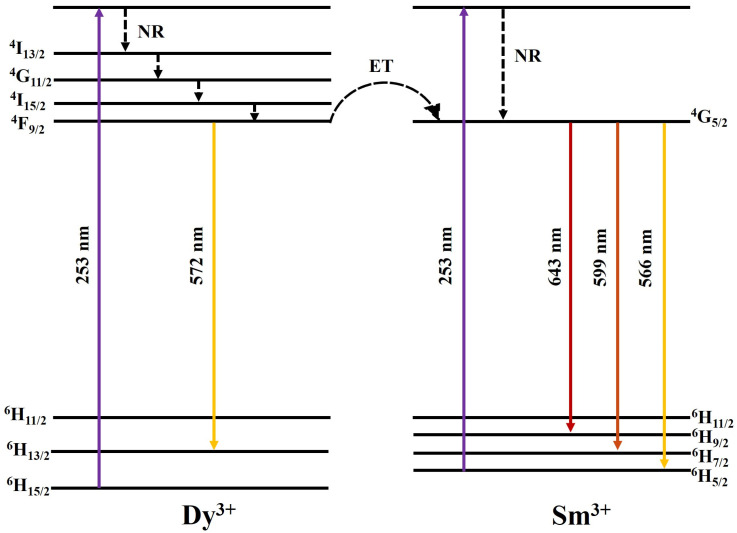
Schematic of energy level structure of the Dy^3+^ and Sm^3+^ ions.

The electrons located at the ground state, ^6^H_15/2,_ of the Dy^3+^ ions absorbed energy under a 253 nm excitation energy and later jumped to the excited state. Since the high energy level was unstable, these electrons dropped successively to the lower-energy excited state, ^4^F_9/2,_ by non-radiative transition (NR). With the populated ^4^F_9/2_ level, the radiative transitions of Dy^3+^ occurred with yellow emissions due to ^4^F_9/2_→^6^H_13/2_ transitions, respectively. In the interim, partial electrons located at the ^4^F_9/2_ level of Dy^3+^ were relaxed to the ^6^G_5/2_ level of Sm^3+^ by the resonance between the two levels, which ultimately gave rise to the characteristic emissions of Sm^3+^ (Figure 8).

### 3.3. Application in a Flexible Composite LED Filter

**Figure 9:**
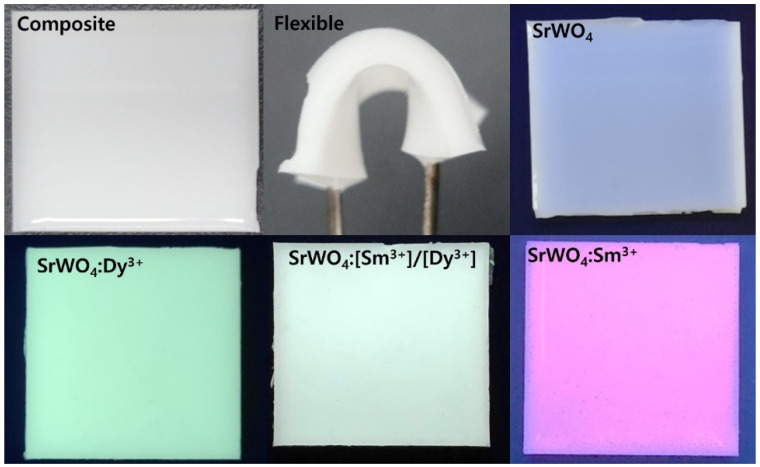
Images of flexible composite under daylight and UV light.

To explore the applicability of the synthesized phosphor, herein referred to as an LED color filter, a flexible composite was fabricated by mixing the phosphor with a PDMS polymer, as explained in detail in Section 2.2. The manufactured composite showed characteristics of blue-white, blue-yellow, and main red light in response to UV light. The composite was flexible by hand and appeared suitable for use as an LED color filter (Figure 9).

## 4. Conclusions

We successfully synthesized crystalline SrWO_4_ at room temperature using the co-precipitation method. When doped with Dy^3+^ and Sm^3+^, yellow and red phosphors were obtained. The crystal structure of the synthesized phosphor was tetragonal, and a change in the lattice constant was observed due to the Dy^3+^ and Sm^3+^ dopants. The synthesized phosphor possessed a cylindrical shape, as confirmed by the FE-SEM images. For the SrWO_4_:[Sm^3+^]/[Dy^3+^] phosphor, the intensity of blue-yellow light emitted by the Dy^3+^ ions decreased as the concentration of Sm^3+^ ions increased. We also fabricated a flexible composite by mixing the synthesized phosphor with a PDMS polymer to demonstrate the potential applicability of the RE-doped phosphor produced in this study. The composite showed blue-white, blue-yellow, and red light emissions under UV and visible light, suggesting its potential application as an LED color filter.

## Figures and Tables

**Table 1:** Reagents and moles used in the synthesis.

SrWO_4_ Up-Conversion Phosphor Synthesis				
Reagents	(CH_3_CO_2_)Sr	Na_2_WO_4_·2H_2_O	Dy(NO_3_)_3_·xH_2_O	Sm(NO_3_)_3_·6H_2_O
Molecular weight (g/mol)	205.93	329.85	348.51	444.47
Used mole (mmol)	1	1	0.25	0.025~0.25
[Dy^3+^]/[Sm^3+^] Ratio				
Reagents	(CH_3_CO_2_)Sr	Na_2_WO_4_·2H_2_O	Dy(NO_3_)_3_·xH_2_O	Sm(NO_3_)_3_·6H_2_O
Used mole (mmol)	1	1	0.25	0.025
	1	1	0.25	0.05
	1	1	0.25	0.075
	1	1	0.25	0.1
	1	1	0.25	0.15
	1	1	0.25	0.2
	1	1	0.25	0.25

## Data Availability

The data presented in this study are available on request from the corresponding author.

## References

[1] Al-Waisawy S., George A.F., Jadwisienczak W.M., Rahman F. (2017). Preparation of balanced trichromatic white phosphors for solid-state white lighting. Luminescence.

[2] Ahn Y.N., Kim K.D., Anoop G., Kim G.S., Yoo J.S. (2019). Design of highly efficient phosphor-converted white light-emitting diodes with color rendering indices (R_1_–R_15_) ≥ 95 for artificial lighting. Sci. Rep..

[3] Wlodarczyk D., Bulyk L., Berkowski M., Glowacki M., Kosyl K.M., Kaczmarek S.M., Kowalski Z., Wittlin A., Przybylinska H., Zhydachevskyy Y. (2019). High-Pressure Low-Temperature Optical Studies of BaWO_4_:Ce, Na Crystals. Inorg. Chem..

[4] Dirany N., McRae E., Arab M. (2017). Morphological and structural investigation of SrWO_4_ microcrystals in relationship with the electrical impedance properties. CrystEngComm.

[5] Koukou V., Martini N., Valais I., Bakas A., Kalyvas N., Lavdas E., Fountos G., Kandarakis I., Michail C. (2017). Resolution Properties of a Calcium Tungstate (CaWO_4_) Screen Coupled to a CMOS Imaging Detector. J. Phys. Conf. Ser..

[6] Sun X., Sun X., Li X., He J., Wang B. (2014). Synthesis and Luminescence of BaWO_4_:Ln^3+^ (Ln = Eu, Tb, and Dy) Powders. J. Electron. Mater..

[7] Huang J., Lu W., Yue D., Wang Y., Wang Z., Jin L., Zhang L., Li Z. (2019). Controllable synthesis of multi-morphological SrWO_4_:Ln^3+^ (Ln = Eu, Tb) hierarchical structures and their luminescence properties. CrystEngComm.

[8] Tian B., Chen B., Tian Y., Sun J., Li X., Zhang J., Zhong H., Cheng L., Hua R. (2012). Concentration and temperature quenching mechanisms of Dy^3+^ luminescence in BaGd_2_ZnO_5_ phosphors. J. Phys. Chem. Solids.

[9] Ofelt G.S. (1962). Intensities of crystal spectra of rare-earth ions. J. Chem. Phys..

[10] Yu M., Xu X., Zhang W., Chen X., Zhang P., Huang Y. (2020). The effect of Sm^3+^ co-doping on the luminescence properties of Ca_2·85_Li_0·15_(PO_4_)_1·85_(SO_4_)_0.15_: Dy^3+^ white-emitting phosphors. J. Alloys Compd..

[11] Jung J., Kim J., Shim Y., Hwang D., Son C.S. (2020). Structure and Photoluminescence Properties of Rare-Earth (Dy^3+^, Tb^3+^, Sm^3+^)-Doped BaWO_4_ Phosphors Synthesized via Co-Precipitation for Anti-Counterfeiting. Materials.

[12] Yi S., Jung J. (2021). Calcium Tungstate Doped with Rare Earth Ions Synthesized at Low Temperatures for Photoactive Composite and Anti-Counterfeiting Applications. Crystals.

[13] Tan X., Wang Y., Zhang M. (2018). Solvothermal synthesis, luminescence and energy transfer of Dy^3+^ and Sm^3+^ doped NaLa(WO_4_)_2_ nanocubes. J. Photochem. Photobiol. A Chem..

[14] Sun X., Huang Z., Fu X., Xu L., Liu K., Yuan H. (2020). Generation of warm white light by doping Sm^3+^ in Ca_3_TeO_6_:Dy^3+^ fluorescent powders. Ceram. Int..

[15] Van Orman J.A., Grove T.L., Shimizu N. (2001). Rare earth element diffusion in diopside: Influence of temperature, pressure, and ionic radius, and an elastic model for diffusion in silicates. Contrib. Mineral. Petrol..

[16] Diaz-Torres L.A., De la Rosa E., Salas P., Romero V.H., Angeles-Chávez C. (2008). Efficient photoluminescence of Dy^3+^ at low concentrations in nanocrystalline ZrO_2_. J. Solid State Chem..

[17] Yasaka P., Kaewkhao J. (2016). White emission materials from glass doped with rare Earth ions: A review. AIP Conf. Proc..

[18] Selvalakshmi T., Sellaiyan S., Uedono A., Chandra Bose A. (2014). Investigation of defect related photoluminescence property of multicolour emitting Gd_2_O_3_:Dy^3+^ phosphor. RSC Adv..

[19] Stojadinović S., Vasilić R. (2016). Orange–red photoluminescence of Nb_2_O_5_:Eu^3+^, Sm^3+^ coatings formed by plasma electrolytic oxidation of niobium. J. Alloys Compd..

[20] Gupta S.K., Pathak N., Kadam R.M. (2016). An efficient gel-combustion synthesis of visible light emitting barium zirconate perovskite nanoceramics: Probing the photoluminescence of Sm^3+^ and Eu^3+^ doped BaZrO_3_. J. Lumin..

[21] Yang L., Mi X., Su J., Zhang X., Bai Z., Wang N., Lin J. (2018). Tunable luminescence and energy transfer properties in YVO_4_:Bi^3+^, Ln^3+^ phosphors prepared by microwave sintering method. J. Mater. Sci. Mater. Electron..

[22] Ayvacikli M., Kaynar Ü.H., Karabulut Y., Canimoglu A., Bakr M., Akca S., Can N. (2020). Synthesis and photoluminescence characteristics of Dy incorporated MoO_3_ phosphor: Suppression concentration quenching. Appl. Radiat. Isot..

